# Investigating the Adipogenic Effects of Different Tissue-Derived Decellularized Matrices

**DOI:** 10.3389/fbioe.2022.872897

**Published:** 2022-04-14

**Authors:** Weiya Tang, Jun Qi, Qian Wang, Yaping Qu, Su Fu, Jie Luan

**Affiliations:** Breast Plastic and Reconstructive Surgery Center, Plastic Surgery Hospital, Chinese Academy of Medical Sciences and Peking Union Medical College, Beijing, China

**Keywords:** decellularized adipose-derived matrix (DAM), acellular dermal matrix (ADM), decellularized matrix, tissue-specific, adipogenesis, tissue remodeling

## Abstract

**Objective:** Decellularized adipose-derived matrix (DAM) can promote adipogenic differentiation and adipose tissue remodeling, but the biological impact of tissue origin on DAM remains unknown. The present study aimed to investigate the effects of tissue origins on the adipogenic capacity of the decellularized matrix by comparing the cellular and tissue responses of DAM versus acellular dermal matrix (ADM).

**Methods:** The *in vitro* response of adipose-derived stem/stromal cells (ADSCs) to DAM and ADM was characterized by proliferation and differentiation. The *in vivo* remodeling response was evaluated in the subcutaneous injection model of immunocompromised mice, using histology, protein expression, and transcriptome analysis.

**Results:** Both DAM and ADM exhibited excellent decellularization effects and cytocompatibility. In the absence of exogenous stimuli, DAM could induce adipogenic differentiation of ADSCs compared with ADM. In the animal model, the levels of PDGF, VEGF, and ACRP30 were higher in the DAM groups than in the ADM group, and more neovascularization and extensive adipose tissue remodeling were observed. The mRNA-seq analysis indicated that the DAM implant regulated tissue remodeling by modulating *Lat1/2* expression along with Hippo Signaling pathway in the early stage.

**Conclusion:** Tissue origin can influence the biological response of the decellularized matrix. DAM can retain favorable tissue-specific characteristics after the decellularization process and have unique adipogenic effects *in vitro* and *vivo*, which can be fully utilized for soft tissue repair and regeneration.

## Introduction

Decellularized matrix can better mimic the tissue microenvironment and promote the directed differentiation of implant cells and tissue remodeling, Compared with scaffolds made of artificial materials (Kim et al., 2020). Due to the removal of immunogenic cellular components, the decellularized matrix has good biocompatibility and safety, which can be widely used in autologous, allogeneic, and xenogeneic tissue engineering.

Compared to other tissues, adipose tissue is widely distributed in the human body and can be obtained in large quantities through fat aspiration and abdominoplasty. The extraction of the decellularized matrix from adipose tissue has extensive application prospects. Previous studies found that decellularized adipose-derived matrix (DAM) could induce differentiation of stem cells to adipocytes and promote adipose tissue remodeling without additional stimulus (Flynn, 2010; Lauren et al., 2019). However, the mechanisms underlying the adipogenic ability of DAM remain unknown, and adipogenic effects of the decellularized matrix from other tissue origins have not been investigated.

Theoretically, decellularized matrices of homologous tissue origin are ideal substrates for cell proliferation and differentiation and tissue functional remodeling. Many studies have concluded that decellularized matrix of homologous tissue origin can maintain a tissue-specific cell phenotype and induce tissue-specific differentiation. When adipose mesenchymal stem cells (ADSCs) were seeded on DAM and decellularized trabecular bone (DTB)respectively, DAM could enhance adipogenic differentiation of ADSCs, while osteogenetic differentiation of ADSCs was more pronounced in DTB (Shridhar et al., 2019). A study compared the cellular response to esophageal extracellular matrix (eECM) versus small intestinal submucosa extracellular matrix (SIS-ECM) and urinary bladder matrix (UBM), and found eECM retains tissue-specific characteristics that enhance the migration of esophageal stem cells and supports the formation of 3D organoids (Keane et al., 2015). Even so, the tissue-specific effect of Decellularized matrix was not significant in some applications, such as DAM was applied to nerve repair (Lin et al., 2011), bone (Clough et al., 2015), and cartilage degeneration (Choi et al., 2012). It can be noted that the tissue specificity of the decellularised matrix is affected by exogenous stimulus and application environments.

As a decellularized matrix that has been widely used in plastic surgery, acellular dermal matrix (ADM) has good therapeutic results in wound repair (Marston et al., 2003; Reyzelman et al., 2009), mammoplasty (Ouyang et al., 2019), cleft lip, and palate repair (Aldekhayel et al., 2012). Different tissue origins of decellularized matrices have different functional compositions, leading to tissue-specific cellular responses. The objective of the present study was to investigate the effects of tissue origins on the adipogenic capacity of the decellularized matrix by comparing the responses of DAM versus ADM *in vitro* and *in vivo*. In this study, DAM and ADM were prepared for co-culturing with ADSCs, which may play a role in adipose remodeling, and cell responses including proliferation and directed differentiation were investigated. The decellularized matrices were then injected into dorsal subcutaneous locations of immunocompromised mice to observe tissue responses and remodeling. In addition, mRNA-seq analysis was used to systematically compare gene expressions of DAM and ADM implants to elucidate underlying signaling pathways in the early stage of adipose tissue remodeling. This study explores the mechanisms that DAM promotes adipose tissue formation and provides further insight into tissue specificity of the decellularized matrix, which could help develop applications for different tissue-derived decellularized matrices in tissue repair and regeneration.

## Materials and Methods

### Preparation of Human Decellularized Adipose-Derived Matrix and Acellular Dermal Matrix

Fresh human adipose tissue was obtained from four healthy female patients, ranging from 30 to 45 years old (average age 38), who underwent abdominal and femoral negative pressure liposuction surgery at the Chinese Academy of Medical Sciences & Peking Union Medical College Plastic Surgery Hospital (Beijing, China). All protocols using human samples were approved by the Plastic Surgery Hospital Ethics Committee (NO.ZX201843), and the samples were obtained with written informed consent.

The human lipoaspirate was added to distilled water and layered after 15 min at room temperature. Remove the oil in the upper layer and blood in the bottom and retain the adipose tissue in the middle layer. Repeat the cleaning several times, and subject the tissue to three cycles (2–3 h each) of freezing and thawing (−80°C to 37°C) in distilled water. Homogenize the adipose tissue at 28,000 rpm for 1 min by a homogenizer (IKA A11, Germany) and repeat three times to realize sufficient homogenization. The suspension was centrifuged at 1,000 rpm for 5 min, the oil in the upper layer was discarded, and the white precipitate in the lower layer was collected. All precipitates were soaked in 0.5 M NaCl (37°C, 4 h), 1.0 M NaCl (37°C, 4 h), distilled water (37°C, overnight), 1% Triton-X100 (37°C, 48 h), distilled water (37°C, 30 min, three times), using a thermostatic shaker (Zhicheng Analytical Instrument, Shanghai, China) at 100 rpm. Next, the precipitates were soaked in 99.9% isopropanol for 8 h to remove the lipid content. Then repeatedly rinsed with distilled water and 75% ethanol three times. The obtained DAM was stored in 1% penicillin-streptomycin solution at 4°C for further experiment.

Human acellular dermal matrix (ADM) was purchased from Beijing Jayyalife Biotechnology Co., Ltd. (Z200713). The ADM and DAM were lyophilized with a vacuum freeze-drying instrument (Xinmozhen Technology (Beijing) Co., Ltd.) and then sterilized with ethylene oxide. The lyophilized matrix was ground with a low-temperature grinder (JXFSTPRP-II-02, Shanghai Jingxin Industry) at a frequency of 70 Hz for 1 min.

### Characterization of Decelluarized Matrices

The samples of fresh adipose tissue, DAM, and ADM were fixed in 4% paraformaldehyde and embedded in paraffin. 5 μm sections were cut for hematoxylin and eosin (HE), masson’s trichrome staining, and picrosirius red staining. The samples were also frozen and stained with oil red O to observe residual oil. In addition, the sample of ADM and DAM were weighed at the same dry weight. Residual DNA and glycosaminoglycans (GAG) were extracted and quantified using Quant-iTTM PicoGreen^®^ dsDNA Reagent and Kits (Thermofisher, United States) and Blyscan Sulfated Glycosaminoglycan Assay (Biocolor, United Kingdom).

### Scanning Electron Microscopy

The inner structure of DAM and ADM was observed using scanning electron microscopy (SEM) (Hitachi SU-8010, Japan). The samples were fixed with 2.5% glutaraldehyde solution at room temperature, followed by gradient ethanol dehydration, isoamyl acetate immersion, vacuum drying, gold spraying, and scanning electron microscopy (SEM) observation.

### Indentation Testing

The mechanical properties of the tissue before and after decellularization were measured by indentation testing. As previously described (Omidi et al., 2014; Li et al., 2022), the specimens of adipose tissue, dermal tissue, DAM, and ADM were cut into disks with a 6-mm diameter (*n* = 5) using a corneal trephine and tested using a 5967 Universal Testing Machine with a 100-N load cell (Instron, Norwood, MA). For unconfined compressive testing, the specimens were compressed at 10 mm/min via the indenters until 100N strain was reached. The yield point was determined, and the yield strength (kPa) was recorded. Young’s modulus was calculated by the slope of linear compression phase of the stress-strain curve.

### Isolation and Culture of Adipose-Derived Stem/Stromal Cells

Sterile adipose tissue was obtained from the liposuction mentioned above. ADSCs were isolated by conventional enzyme digestion (0.5 mg/ml collagenase I, 50 min, 37°C). The primary ADSCs were cultured with low-glucose Dulbecco’s Modified Eagle’s Medium (10% fetal bovine serum, 1% penicillin, and 1% streptomycin) at 37°C with 5% CO_2_ in a humidity atmosphere. Change the culture medium every 2 days and passage the cells after 70%–80% confluence. The third-forth passage ADSCs were chosen for further experiment. The complete growth medium was replaced with the differentiation medium (including 0.25 mM isobutylmethylxanthine and 1 mg/ml of troglitazone) to induce differentiation.

### Cell Seeding

The lyophilized matrix was cut into 10 mg pieces then were rinsed in 75% ethanol and sterile PBS. 60 μl ADSCs suspension (10 (Keane et al., 2015) cells) were seeded in the DAM and ADM and placed into 24-well transwell culture inserts in a 0.4 μm layer. After 24 h in culture, the ADSC-matrix composites were transferred to a new 24-well plate for better exposure to the medium.

Cell viability and adhesion rate were determined using the PrestoBlue™ Cell Viability Reagent (Thermo, United States). An equal number of cells were seeded in blank wells as the control group in the 24-well plate (*n* = 3 for each group). PrestoBlue solution (400 μl) was added to each well, and the plates were incubated at 37°C for 30 min. After incubation, the fluorescence intensity with excitation wavelength at 560 nm and emission wavelength at 590 nm was measured using a microplate reader (Multiskan, Thermo Fisher Scientific, United States). The LIVE/DEAD^®^ Viability/Cytotoxicity Assay Kit (Thermo Fisher) was used to visualize the attachment and stretching of cells at days 2 and 5 after cell seeding. Confocal microscopy was performed using a Leica TCS SPII microscope (Leica, Allendale, NJ).

### Quantitative Real-Time Polymerase Chain Reaction

Gene expression of adipogenic marker (PPARγ) was characterized by qRT-PCR analysis. Total RNA of samples at days 7 and 14 were extracted using TRIzol reagent (Invitrogen, Burlington, VT, Canada) from samples following the manufacturer’s protocol. Primer sequences of PPARγ is (5′–3′) F: TGG​AAT​TAG​ATG​ACA​GCG​ACT​TGG R: CTG​GAG​CAG​CTT​GGC​AAA​CA. The RNA template was converted into cDNA using SYBR Green Master Mix (Life Technologies). Quantitative reverse-transcription polymerase chain reaction (qRT-PCR) was conducted on a StepOnePlus Real-Time PCR System. The amplification was performed using the cycling conditions- 3 min at 95°C, followed by 40 cycles of 15 s at 95°C and 20 s at 60°C. All samples were tested for three biological replicates. The gene expression level was determined according to the fluorescence signal and calculated by the 2^−ΔΔct^ method, using GAPDH as an internal reference gene standardization.

### Experimental Animal Model

The immunocompromised mice used in the experiment were C57Bl/6 (B6.129S7-Rag1^tm1Mom^/J) female mice aged 6–10 weeks (Shanghai Model Organisms Center Inc., Shanghai, China). The animal experiment protocol strictly followed the regulations and standards of protection and use of experimental animals set forth by the Chinese Academy of Medical Sciences & Peking Union Medical College.

After grinding, the DAM and ADM powders were weighed, and 0.4 ml of normal saline was added into 5 mg of the powders to mix thoroughly. The injection area on the dorsal skin was shaved and aseptically prepared. 0.4 ml matrix suspension was injected into the back of mice per side using 18-gauge needles. So each mouse could get two separate samples. Mice were euthanized at weeks 1, 3, 5, 8, and 12 after injection, and samples of the implants were then obtained.

### Histological and Immunohistochemical Staining

After being fixed in the 4% paraformaldehyde for 24–48 h, samples were paraffin-embedded and sectioned (5 μm sections). Haematoxylin and eosin (HE) and Masson’s trichrome staining were used to examine the morphology of the implants at each study time point. Immunohistochemistry against the adipogenic marker perilipin-1 (Abcam ab3526) was performed. In addition, CD31 (Proteintech 28083-1-AP) was used to localize vascular endothelial cells. In order to compare the observed adipogenesis between DAM and ADM, the adipose area was measured across the entire implant cross-section and expressed as a percentage of the total implant area for all time points. Angiogenesis in implants was assessed by measuring the percentage of CD31-positive areas as also. All analyses were conducted in a blinded fashion in 5–10 randomly selected and non-overlapping fields across the implant using Olympus BX51 microscope (Olympus, Center Valley, PA) and Image Pro Plus 6.0.

### The mRNA-Seq Analysis

Total RNA was extracted from tissues of DAM and ADM implants at 1 week using standard protocols. The sample size for conventional mRNA-seq libraries was fixed at three biological replicates. After the qualifications of RNA samples, the common transcriptome libraries were constructed. Qubit 3.0 was used for preliminary quantification, and qPCR was used to quantify the effective concentration of the library accurately. After the library inspection, PE150 mode sequencing was performed using Illumina NovaSeq 6000 sequencing platform. The obtained sequencing datum was qualified, then these high-quality sequences were aligned to the reference genome. Differential expression significance analysis was performed using edgeR, and the actual analysis parameters used in this analysis were: |log2(Fold Change) | > 1, qvalue < 0.05. Enrich-KEGG (Kyoto Encyclopedia of Genes and Genomes) method and Enrich-GO method were utilized to calculate enrichment test for KEGG pathways and Gene Ontology terms.

The sequencing results were verified using the qRT-PCR method described above. The primer sequences are listed in Supplementary Table S1.

### Cytokine Assessment by ELISA and Luminex Assay

After injection for 1, 3, and 5 weeks, the implants were harvested entirely to detect the level of related factors. Each sample was added 300–500 μl of the lysate (abs9225, Absin, China), then was ground at low temperature. Protein suspensions were obtained by centrifugation at 14,000 rpm, 4°C for 10 min after 2 repetitions. The protein concentration in each sample was measured by the BCA method (Beyotime, China). Assays were performed as per the manufacturer’s instructions (*n* = 3).

ELISA kits were used to assess acrp30 (R&D Systems) and TNF-a (absin) levels. Samples and standards were added to each well of the plate. Plates were incubated for 2–3 h at room temperature (R.T.) and rinsed 4 times with wash buffer, then the buffer was removed and 200 μl conjugate was added. Plates were incubated for 1–2 h at R.T., rinsed 4 times, the buffer removed, and 100 ml color substrate added. After incubating 30 min in the dark at R.T., 50–100 μl stop solution was added to each well. Plates were read using a Multiskan microplate reader (Thermo) set at 450 nm absorbance with a correction reading set at 570 nm, and values were calculated back to pg/ml of the original sample volume.

The Luminex assay was used to assess CCL2/JE/MCP-1, CCL3/MIP-1 alpha, FGF basic/FGF2/bFGF, IFN-γ, IL-1 beta/IL-1F2, IL-4, IL-6, IL-10, PDGF-AA, and VEGF levels. Prepare all the required reagents and samples. Add 50 μl of diluted beads and 50 μl of standards or samples to each well, shake for 2 h at R.T., place the plate on a magnetic stand to ensure the beads are held in place, and wash three times with washing solution. 50 μl of antibody complex was added to each well and shook for 1 h, 800 rpm at R.T. The plate is then placed on a magnetic stand to ensure that the beads are held in place and washed three times with washing-up liquid. Add 50 μl of streptavidin-labeled P.E. to each well and shake for 0.5 h at R.T. Place the plate on a magnetic stand to ensure that the beads are held and wash three times with washing solution. The beads were resuspended with 100 μl of washing solution, incubated, and shook for 2 min with the speed of 800 rpm. The plate was tested on the Luminexv (X-200).

### Statistical Analysis

All data are expressed as the mean ± standard deviation (SD). Statistical analyses were performed using Prism 9.0 software (Graphpad Software, United States). The statistical significance of data was analyzed using analysis of variance (ANOVA) at the 95% confidence level. *p* < 0.05 was considered statistically significant (*p < 0.05, **p <0.01, ***p < 0.001, ****p < 0.0001).

## Results

### Evaluation of Decellularized Adipose-Derived Matrix and Acellular Dermal Matrix

**FIGURE 1:**
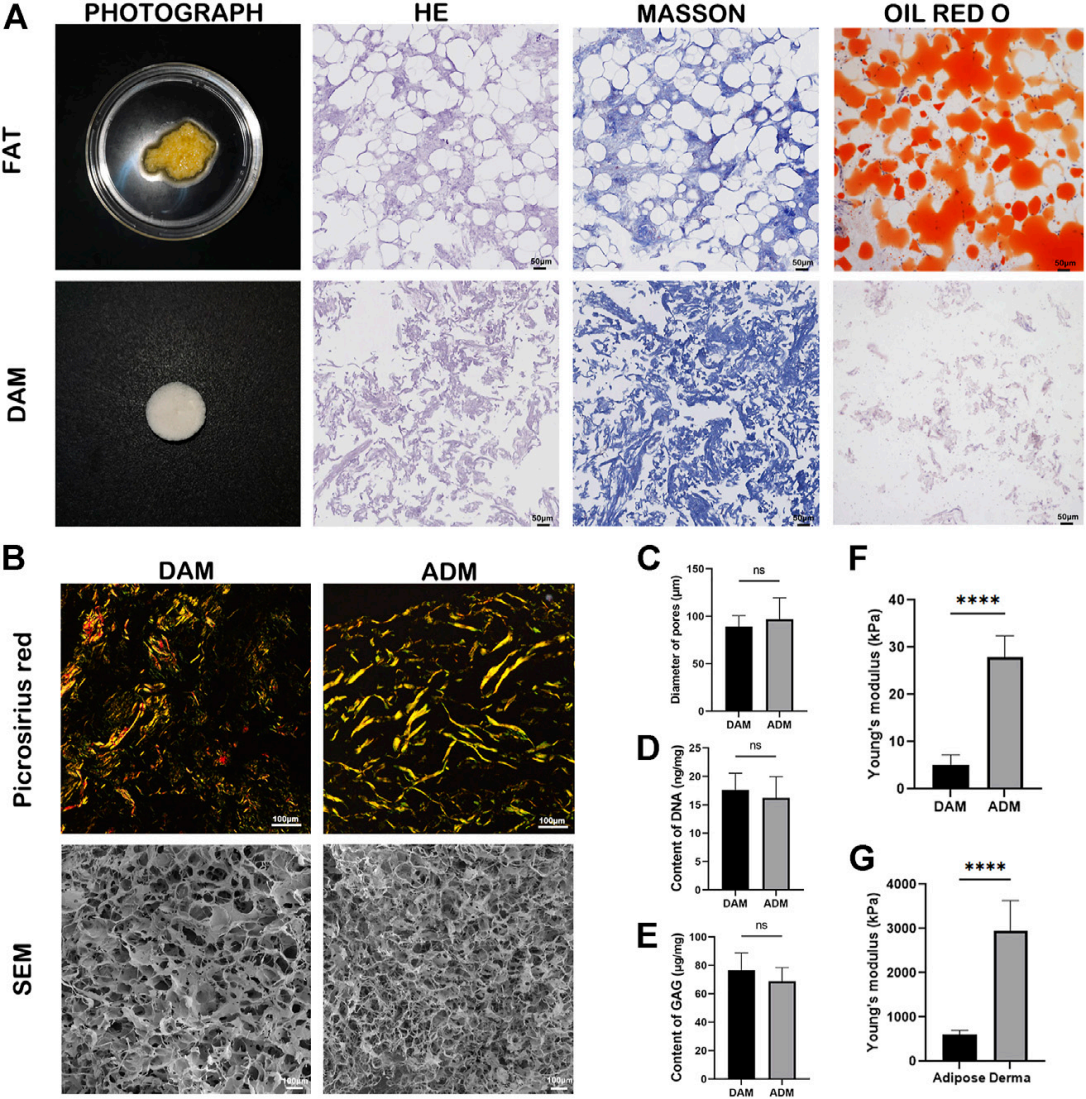
Characterizations of DAM and ADM. **(A)** Photograph, H&E staining, Masson’s Trichrome and Oil-red O staining of adipose tissue and DAM. Scale bars = 50 μm. **(B)** Picrosirius red staining and SEM of the DAM and ADM. Scale bars = 100 μm. **(C)** Diameter of DAM and ADM pores in SEM, *n* = 5. **(D)** The content of DNA in DAM and ADM, *n* = 5. **(E)** The content of GAG in DAM and ADM. *n* = 5, ns, no significance. **(F)** The Young’s modulus of DAM and ADM. *n* = 5, ****p < 0.0001. **(G)** The Young’s modulus of adipose and derma. *n* = 5, ****p < 0.0001.

Physical and chemical treatments were used to realize the decellularization of adipose tissue. Macroscopically, DAM showed a white, homogeneous, and loose appearance (Figure 1A). H&E staining confirmed the absence of cells and cell debris in DAM at the end of the process (Figure 1A). Masson’s Trichrome staining showed blue-stained collagenous components without red staining nuclei, which also confirmed the effectiveness of the decellularization process (Figure 1A). Oil-red O staining showed no red-stained lipid components, indicating that the lipid components in adipose tissue were effectively removed (Figure 1A).

Picrosirius red staining (Figure 1B) was used to compare the distribution and organization of collagens in the DAM and ADM. Both decellularized matrices contained tightly arranged yellow or red type I collagen, a small amount of green type III collagen, and yellowish type IV collagen. The DAM was arranged in a relatively loose network consisting of a blend of thick and thin fibers. While in ADM, it was arranged in a dense woven pattern with thicker fibers (Figure 1B). SEM micrographs depicted the inner microstructure of DAM and ADM (Figure 1B) also confirmed the removal of cells from the tissue. After grinding, the overall ultrastructure of DAM and ADM were similar with pores and entwined fibers structure. In addition, the pores of DAM were more evenly distributed with the thicker and more orderly fibers. ADM had larger pores (96.80 ± 22.64 μm) than DAM (89.00 ± 11.73 μm) with no significance (Figure 1C).

The content of DNA and GAG were detected in prepared DAM, taking commercial ADM as a comparison (*n* = 5). The residue of DNA in the DAM group was 17.60 ± 2.97 ng/mg, and that in the ADM group was 16.20 ± 3.77 ng/mg (Figure 1D). The GAG content in the DAM group was 76.6 ± 12.18 μg/mg, and that in the ADM group was 68.80 ± 9.68 ng/mg (Figure 1E). There were no significant differences in DNA and GAG content in the two groups. The results showed that the acellular method could effectively remove cellular components and retain active ingredients of the decellularized matrix.

The results of indentation testing showed that Young’s modulus measured for each group of samples were: derma (2,952.00 ± 678.80 kPa)> adipose (604.20 ± 96.59 kPa)> ADM (27.94 ± 4.48 kPa)> DAM (5.17 ± 2.11 kPa) (Figures 1F,G). Young’s modulus of Dermal tissue and ADM were significantly higher than that of adipose tissue and DAM, respectively. Although ADM is closer to adipose tissue in terms of Young’s modulus values, it is important to note that fresh tissue is a composite structure containing multiple components compared to decellularized matrix, so it is not suitable to directly compare Young’s modulus of both.

### The Viability and Differentiation of ADSCs Seeded on Decelluarized Matrices

**FIGURE 2:**
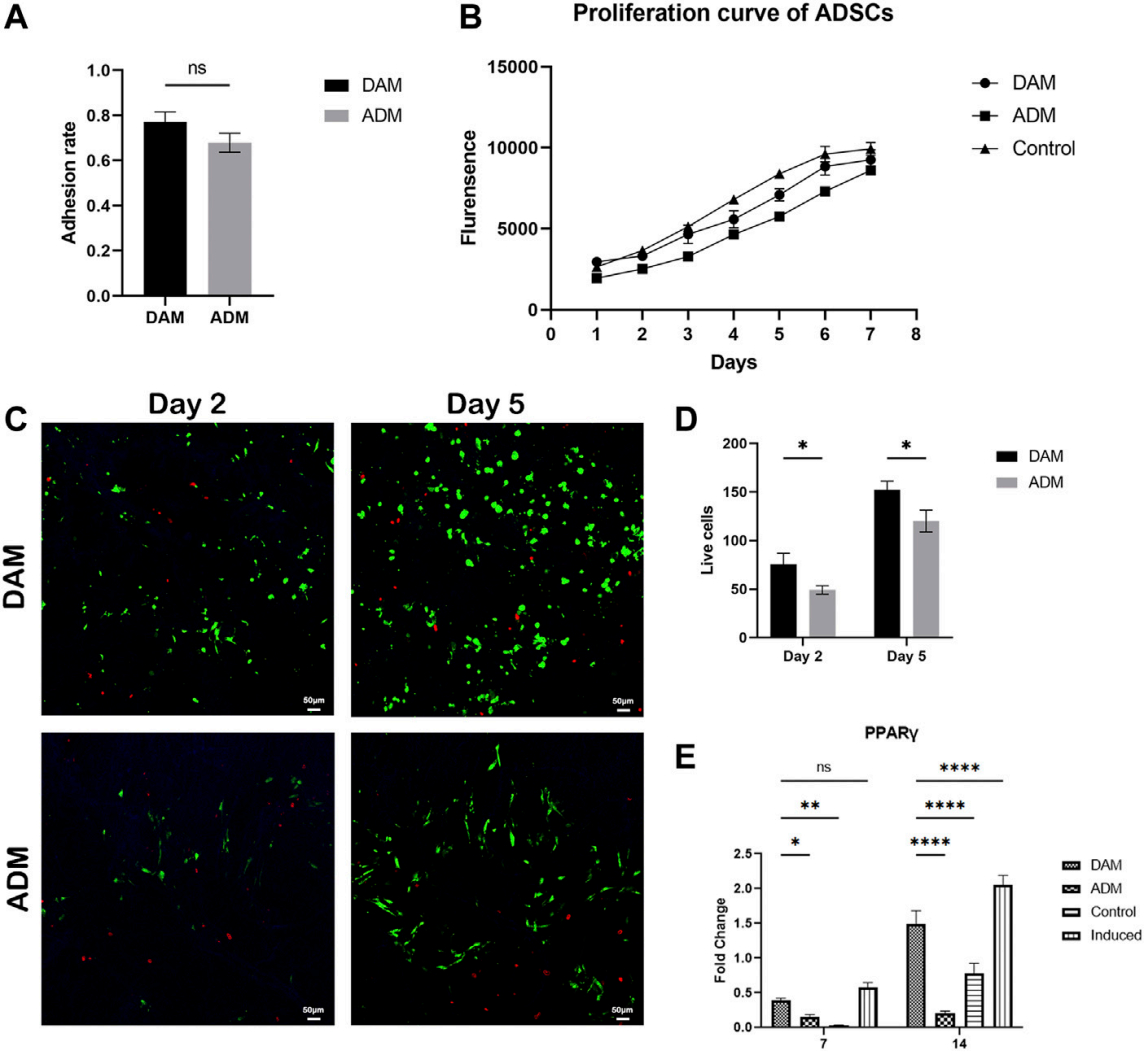
The viability and differentiation of ADSCs seeded on DAM and ADM. **(A)** Adhesion rate of ADSCs to DAM and ADM at 24 h after being seeded, *n* = 5. **(B)** The proliferation and differentiation of ADSCs seeded on DAM and ADM. **(C)** DAM or ADM co-cultured with ADSCs stained by LIVE/DEAD^®^ assay at days 2 and 5. Scale bars = 50 μm. **(D)** The number of green fluorescence-labeled living ADSCs in DAM and ADM, *n* = 5. **(E)** Adipogenic gene *PPARγ* expression of ADSCs in DAM, ADM, control, and induced groups at days 7 and 14. *n* = 3, ns, no significance, *p < 0.05, **p < 0.01, ****p < 0.0001.

After ADSCs were seeded in DAM and ADM for 24 h, 77.04 ± 4.46% and 67.94 ± 4.21% of the cells successfully adhered to the DAM and ADM, respectively (Figure 2A). The proliferation curve of the ADSCs changed slightly compared with the control group, but the cells remained in a good growth state (Figure 2B). Confocal imaging demonstrated that calcein AM–stained ADSCs attached and infiltrated the acellular matrix (Figure 2C). From days 2–5, the number of green fluorescence-labeled living cells increased from 75.8 ± 11.08 to 152.2 ± 8.96 in DAM and 49.2 ± 4.38 to 120 ± 11.18 in ADM, with few red fluorescence-labeled dead cells (Figure 2D). Also, live ADSCs are more numerous and evenly distributed in the DAM than in the ADM.

Quantitative RT-PCR analysis of adipogenic gene revealed that the DAM group and induced group (ADSCs were cultured in differentiation medium) exhibited a higher expression level of *PPARγ* and compared to that on ADM at days 7 and 14. The levels of adipogenic maker were not detectable found in the control group (ADSCs were cultured in growth medium), which were relatively low in the ADM group. The expression of the critical regulator of adipogenesis in the non-induced DAM group suggested the microenvironment was conducive to adipogenesis, while the ADM group was not (Figure 2E).

### Tissue Response and Implant Remodeling

**FIGURE 3:**
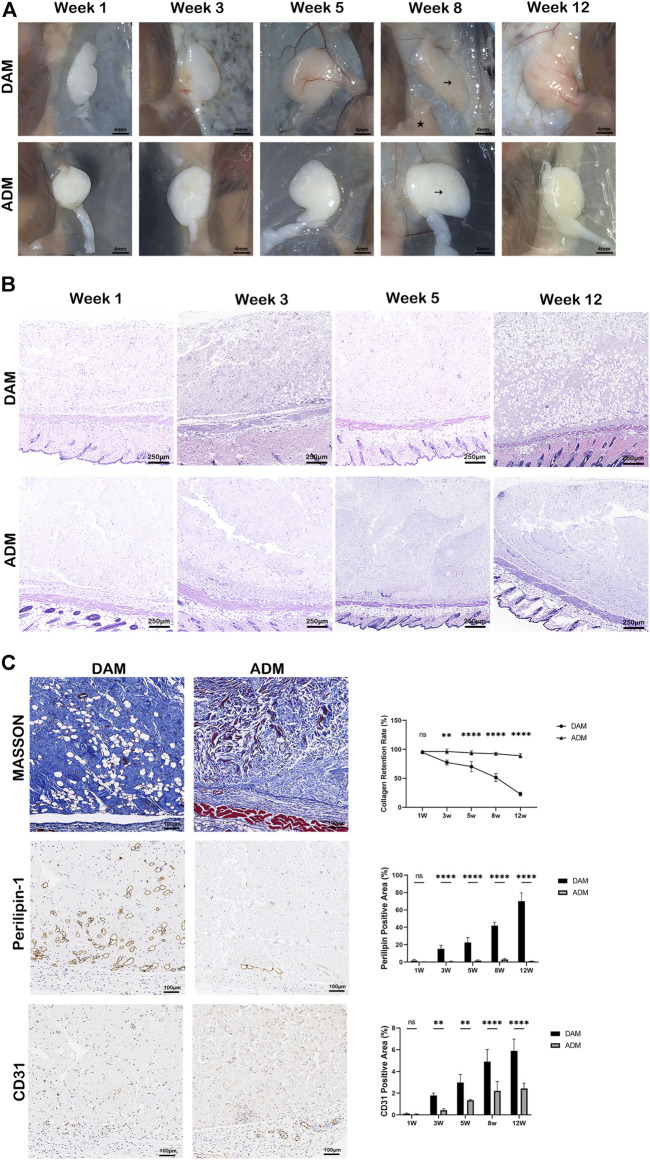
Histology and immunolabeling of DAM and ADM after injection. **(A)** Visual observation of implants at weeks 1, 3, 5, 8, and 12. The DAM and ADM implants (black arrows) were adjacent to mice’s subcutaneous adipose tissue (pentacle). Scale bars = 4 mm. **(B)** H&E staining of DAM and ADM implants at weeks 1, 3, 5, and 12. Scale bars = 250 μm. **(C)** Masson trichrome staining and Immunohistochemistry of implants at weeks 5. Collagen retention rate and percentage of perilipin-1 and CD31 positive areas at different time points were displayed. Scale bars = 100 μm, *n* = 5, ns, no significance, *p < 0.05, **p < 0.01, ****p < 0.0001.

In order to compare the response of decellularized matrix from different tissue sources implanted *in vivo*, DAM and ADM were injected subcutaneously into the back of immunocompromised mice, and samples were harvested at 1, 3, 5, 8, and 12 weeks after injection. Based on visual observation, the two groups of implants formed a complete capsule on the surface, with clear boundaries with surrounding tissues (Figure 3A). The color of implants in the DAM group gradually changed from white to fleshy pink, and the texture was softer, similar to the surrounding subcutaneous adipose tissue. In contrast, the implant in the ADM group always maintained a white appearance with a hard texture. As seen in HE staining, scaffold cellularity was enhanced in the DAM group, particularly at the 3, 5, and 12 weeks (Figure 3B; Supplementary Figure S1). At 3 weeks, adipocytes aggregated from the edge of the implant to the center in the DAM group, and then, the number of adipocytes increased and gradually occupied the implant at 5–12 weeks. In contrast, infiltrating cells were significantly reduced in the ADM group, and almost no adipocytes were observed. Specifically, most implant regions in the DAM group had been remodeled into mature adipose tissue at 12 weeks.

Masson trichrome staining was used to compare collagen retention and the tissue remodeling between two groups (Figure 3C; Supplementary Figures S2, S3). The collagen areas were stained as blue and expressed as a percentage of the total implant area at all time points. At 1 week after injection, minimal remodeling was observed in both groups. At 3 weeks, the percentage of collagen was 77.39 ± 4.25% in the DAM group compared to 96.48 ± 4.13% in the ADM group. This difference was enhanced at 5 and 8 weeks, 70.32 ± 8.92% and 51.50 ± 6.95% of the collagen retention were in the DAM group, compared with 93.90 ± 3.54% and 92.48 ± 2.34% in the ADM group separately. Up to week 12, only 22.99 ± 3.21% of the DAM implant had not been remodeled, while 89.03 ± 3.66% of the ADM implant was still retained.

Immunohistochemistry with antibodies against perilipin-1 revealed positive expression of adipocyte. The percentage of positive perilipin-1 areas was used to compare adipogenesis between two groups at different time points. The trend of the data was opposite to that of collagen retention above. The less collagen left, the more adipose tissue was remodeled. At week 1, no apparent adipocytes were observed in both groups without significant difference. From weeks 3–12, the percentage of adipocytes area ranged from 1.83 ± 1.21% to 70.16 ± 9.50% in the DAM group. While in the ADM group, only a small amount of adipocytes could be observed at weeks 5 (1.76 ± 0.88%) and 8 (3.68 ± 1.85%), which was dropped at weeks 12 (1.08 ± 0.23%). Overall, steady adipogenesis was observed in the DAM group, while rare and unstable in the ADM group (Figure 3C; Supplementary Figures S2, S3).

Angiogenesis in implants was assessed by the percentage of CD31 positive area of the total implant area. The constant growth of neo-blood vessels could be seen in both groups as time went by. At 3, 5, 8, and 12 weeks, blood vessel densities in the DAM group were higher than the ADM group with significant differences. It was also noted that adipogenesis is inevitably accompanied by angiogenesis in the DAM group (Figure 3C; Supplementary Figures S2, S3).

### Cytokine and Adipokine Levels in Implants

**FIGURE 4:**
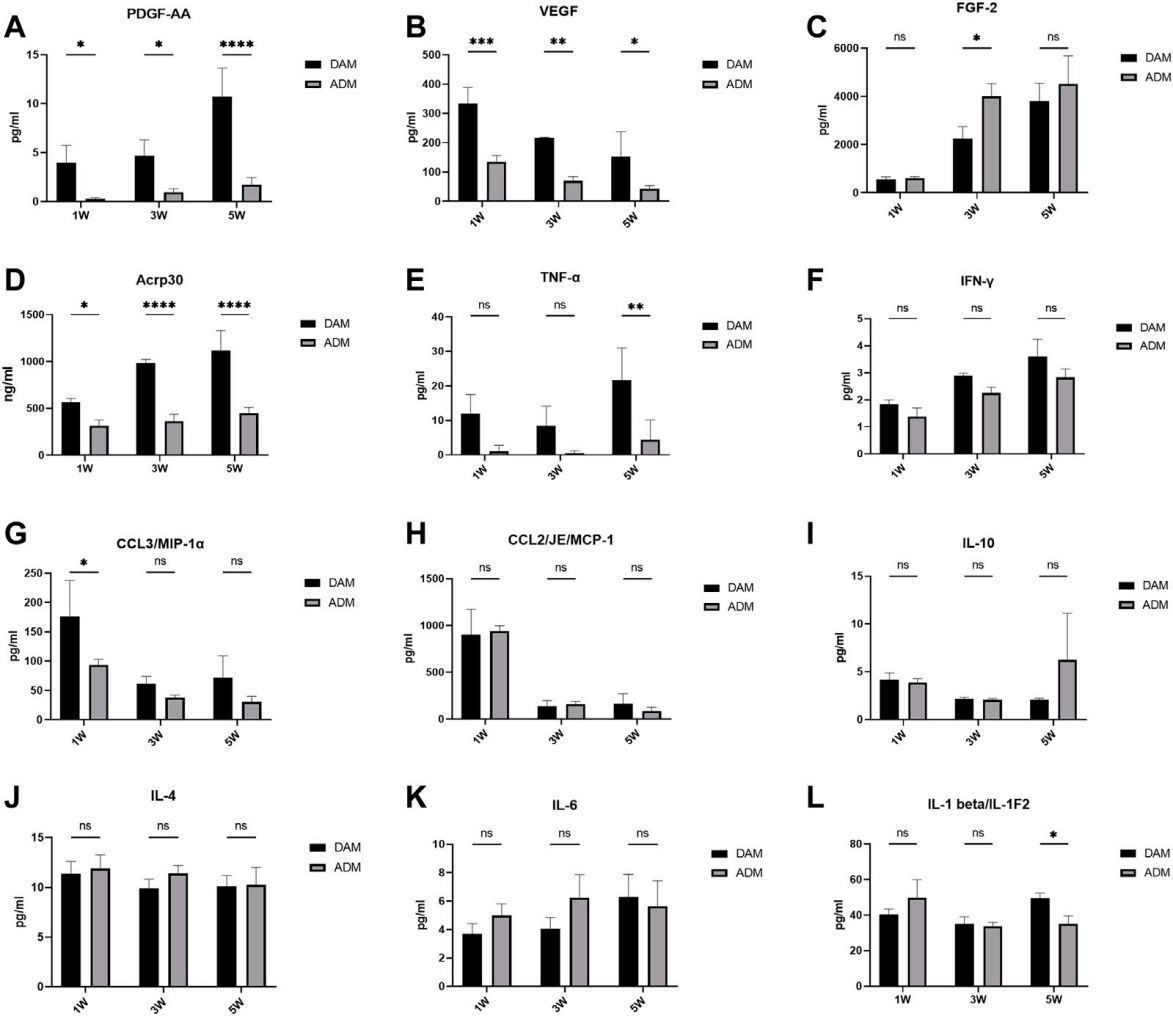
Cytokine and adipokine levels in DAM and ADM implants. *n* = 3. ns, no significance, *p < 0.05, **p < 0.01, ***p < 0.001, ****p < 0.0001.

PDGF and VEGF in the implants remained at higher levels in the DAM group than in the ADM group. There was a gradual increase in PDGF levels and a gradual decrease in VEGF levels over time in both groups (Figures 4A,B). FGF-2 and Acrp30 were measured at significantly higher levels than other cytokines and constantly increased from 1 to 5 weeks. FGF-2 levels were significantly higher in the ADM group than in the DAM group at week 3 (Figure 4C). As an adipose marker mainly secreted by adipocytes, Acrp30 was significantly more in the DAM group than in the ADM group, consistent with the histological observation of adipogenesis (Figure 4D).

Related inflammatory cytokines levels were also measured. The level of TNF-α remained consistently low in the ADM group and was significantly lower than in the DAM group at week 5 (Figure 4E). DAM groups had higher IFN-γ levels than ADM groups with no significance and an upward trend over time in both (Figure 4F). Both MCP-1 and MIP-1α levels remained relatively high in the first week, with MIP-1α levels in the DAM group being significantly higher than in the ADM group, and a substantial number of macrophages were also observed within the implants in the first week, followed by significant declines in weeks 3 and 5 (Figures 4G,H). The levels of IL-10, IL-4, IL-6, and IL-1β fluctuated over time, with no significant differences between the two groups, except at weeks 5, when IL-1β levels in the DAM group were higher than that in the ADM group (Figures 4I–L). Although IL-6 levels in the DAM group were lower than in the ADM group at weeks 1 and 3 with no significance, as the number of adipocytes in the DAM increased and could also secrete IL-6, this might lead to higher levels of IL-6 in the DAM than in the ADM at weeks 5 (Figure 4K).

### Transcriptome Profiling With Functional Enrichment Analysis

**FIGURE 5:**
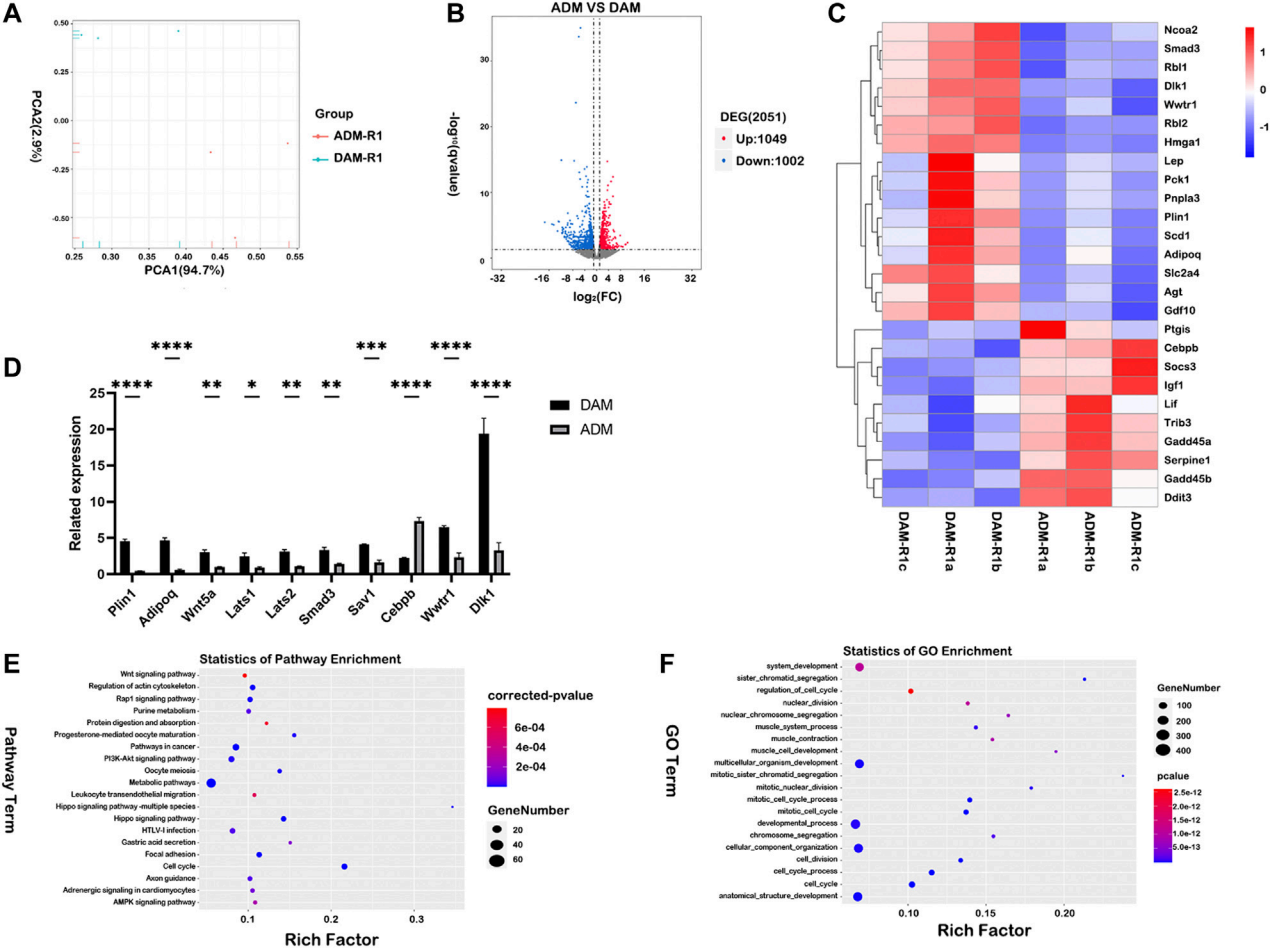
Comparative transcriptome profiling of DAM and ADM implants using functional enrichment analysis. **(A)** Principal component analysis (PCA) was used to show potential similarities between the two groups. **(B)** Differentially expression genes (DEGs) of the two groups showed that a total of 2,051 genes were differentially expressed, of which 1,049 genes were upregulated and 1,002 genes were downregulated. **(C)** Adipogenesis-related genes were selected from DEGs and listed as heatmaps. **(D)** The qRT-PCR analysis verified the results of the microarray analysis. *n* = 3. *p < 0.05, **p < 0.01, ***p < 0.001, ****p < 0.0001. **(E)** The top-20 upregulated KEGG pathways in the DAM group were listed based on DEGs. **(F)** The top-20 upregulated GO terms (Biological Process) in the DAM group were listed based on DEGs.

Histology indicated DAM and ADM implants generated different tissues remodeling *in vivo*. In order to further investigate the molecular mechanisms for gene regulatory networks in the early stage after injection, mRNA-seq analysis was performed for samples harvested on day 7 (*n* = 3 for each group). Principal component analysis was used to show potential similarities between the two groups (Figure 5A). Differentially expression genes (DEGs) of the two groups showed that a total of 2,051 genes were differentially expressed, of which 1,049 genes were upregulated and 1,002 genes were downregulated (Figure 5B). The result confirmed that the DAM induced different tissue responses compared with ADM. Adipogenesis-related genes (Dlk1, Rbl2, Hmga1, Plin1, Smad3) were selected from DEGs and listed as heatmaps (Figure 5C). Most of the genes were up-regulated in the DAM group at week 1, which might play an essential role in the early stage of adipose tissue remodeling, and the reliability of the transcriptome analysis was verified by qRT-PCR (Figure 5D).

Furthermore, gene expression enrichment of several signal pathways using KEGG enrichment analysis was investigated to understand the specific responses observed in different groups. The top-20 upregulated and downregulated KEGG pathways were listed based on DEGs in the DAM and ADM groups (Figure 5E; Supplementary Figure S4). Overall, the gene enrichment pathways Hippo signaling pathway, PI3K-Akt signaling pathway, and Rap1 signaling pathway, which were upregulated in DAM, were all associated with lipid metabolism and adipogenesis (Figure 5E). In contrast, the listed top-20 upregulated KEGG pathways in the ADM group indicated a large variation between the two decellularized matrices in tissue responses (Supplementary Figure S4). Interestingly, metabolic pathways were enriched in both groups with different subpathways.

Analysis of functional enrichment was applied in gene ontology (GO) terms. (Figure 5F) At the initial phase of DAM implantation, up-regulated genes involved in cell proliferation (GO:1903047, GO:0022402) and tissue regeneration (GO:0048856, GO:0032502) contained more up-regulated gene expression. Besides, protein targeting (GO:0006614, GO:0045047) is more concentrated in the ADM group (Supplementary Figure S5).

## Discussion

In previous studies, multiple types of tissues have been decellularized for various applications (Badylak et al., 2009; Sadtler et al., 2016; Dziki and Badylak, 2018). It remains unclear how different tissue-derived decellularized matrices produce different tissue properties and subsequently cause specific tissue responses. Adipose tissue is more accessible than other tissues (heart, liver, bladder, etc.) and can be an important source of the decellularized matrix. Studies on the tissue specificity of the decellularized matrix will help achieve functional regulation of decellularized matrix and facilitate clinical translation. The present study shows that the decellularized matrix of homologous tissue origin-DAM enhances adipogenic differentiation of ADSCs *in vitro* and promotes adipose tissue remodeling *in vivo*. These results suggest that the tissue-specific properties of adipose tissue are retained after the decellularization process and can be identifiable in the immunocompromised mouse model.

The cell and tissue responses to the decellularized matrix are influenced by many factors, such as the decellularization process, the sterilization method, and the tissue source. Different decellularization methods are chosen because each tissue has different physical properties (Crapo et al., 2011). Therefore, it is also difficult to standardize the method of decellularization for different tissues. Decellularization methods usually include physical, chemical and biological methods, and more than two methods are used together in the processing (Dong et al., 2018). In this study, physical methods combined with chemical methods were used to decellularize the obtained adipose tissue of human origin, taking advantage of adipose tissue’s soft and fragile nature. Firstly breaking the adipose tissue by freeze-thawing cycles and grinding can increase the contact area of reagents and improve the decellularization efficiency. Then the cellular components were removed with a milder non-ionic detergent (1% Triton X-100), thus reducing damage to the tissue structure and composition by chemical detergents (Du et al., 2011). Finally, the oil was removed with isopropyl alcohol in a shorter period, which is more conducive to maintaining the natural properties of DAM (Brown et al., 2011). No biological enzymes were used in the experiment, compared with the traditional decellularization method (Flynn, 2010), which can avoid the damage led from enzymes to the matrix structure and reduce the exogenous contamination.

Considering the different textures and compositions of dermal and adipose tissues, clinically available human-derived ADM was purchased directly for the experiment. The properties of the two decellularized matrices obtained are compared in histological staining, microstructure by electron microscopic scanning, and DNA and GAG content. The comparison results demonstrated the validity of the decellularization method employed in the experiments. To facilitate injection and use, many researchers have prepared the decellularized matrix in the form of the hydrogel by enzymatic digestion or polymer material compounding (Wu et al., 2012; Adam Young et al., 2014; Brown et al., 2015). However, we would like to preserve the original state of the decellularized matrix as much as possible and reduce the interference of exogenous factors. Therefore, the two decellularized matrices were ground, and the microstructures of DAM and ADM were similar in scanning electron microscopy, showing a sparse structure with uniform distribution of pores.

Our study demonstrated *in vitro* that DAM provides an inductive microenvironment for adipogenic differentiation of ADSCs. PPARγ is considered the master controller of adipogenic differentiation (Morrison and Farmer, 2000). ADSCs expressed higher levels of PPARγ in the complete medium after seeding in DAM, compared to the ADM group and blank group. There is growing evidence that decellularized matrices can help induce stem cell differentiation *in vitro*. In addition to DAM, DTB can also promote osteogenic differentiation of stem cells without induction factors (Hung et al., 2016; Paduano et al., 2017). Researchers found that DAM showed enhanced adipogenic induction of ADSCs in the adipogenic induction medium, while in the osteogenic induction medium, DTB showed enhanced osteogenic induction (Shridhar et al., 2019). It is important to be noted that ADSCs exhibit differentiation consistent with the induced differentiation medium even in different decellularized matrices. This suggests that the tissue specificity of the decellularized matrix does exist but is also susceptible to exogenous factors. In addition, Young’s modulus of DAM was significantly lower than that of ADM, which was also more favorable to the lipogenic differentiation of ADSC in cellular experiments. Previous studies have shown that tissue stiffness is an important factor contributing to the direction of stem cell differentiation (Levy-Mishali et al., 2009; Gilpin and Yang, 2017), and lower stiffness is more favorable for adipogenic differentiation of stem cells compared to osteogenic differentiation (Marinkovic et al., 2016). Currently, Young’s modulus and tensile strength are the most common measurements to study the mechanical properties of DAM. However, the relevant data obtained from multiple studies were not similar and fluctuated over a wide range (Yu et al., 2013a; Omidi et al., 2014), which might be due to the differences in the decellularization method, the physical state, and the measurement method of each study. For these reasons, there is a lack of uniform standards for the mechanical properties of various forms of decellularized matrices.

The environment *in vivo* is more complex than that *in vitro*, and the remodeling reaction of the decellularized matrix *in vivo* is closely associated with tissue regeneration. The DAM implant significantly enhanced adipogenesis in immunocompromised mice in the current study. Adipogenesis is accompanied by collagen degradation and angiogenesis in the decellularized matrix. By Masson staining, it is evident that ADM and DAM have different degradation rates *in vivo*. In fact, until week 12, ADM can maintain more than 80% of collagen residues after subcutaneous injection, which is only about 20% in DAM. The decellularized matrix releases bioactive peptides during degradation and plays a vital role in tissue remodeling. It can initiate and enhance the remodeling process, such as angiogenesis (Li et al., 2004), mitogenesis, and chemotaxis of site-specific cells (Agrawal et al., 2009), and can recruit progenitor cells (Agrawal et al., 2010). The different degradation rates of DAM and ADM may also contribute to the different responses *in vivo*. When ADM combined with tissue expander was used in breast reconstruction surgery, it was found that after several months *in vivo*, ADM occurred with little degradation and local cellular infiltration (Gaster et al., 2013). Although ADM had been ground and injected into the mice, 12 weeks later, it still indicated little degradation, which may be related to the composition of ADM. The decellularized matrix is usually composed mainly of collagen, laminin, elastin, and GAG, including various cytokines (Marçal et al., 2012; Aamodt and Grainger, 2016). The types and amounts of these proteins in decellularized matrices of different tissue origins are not the same, which may affect the cellular responses of the decellularized matrix. However, it is still challenging to determine which protein differences are responsible due to the impacts of different methods for decellularization and protein extraction (He et al., 2019; Jiang et al., 2021).

The development of mature adipose tissue requires the support of an extensive capillary network (Cao, 2007). The neovascularization occurred and grew over time in both decellularized matrices implants, which was more pronounced in the DAM. ADM has been studied and used for a longer period than DAM, and neovascularization has been found in ADM grafts in both clinical trials and animal studies (Gaster et al., 2013; Boháč et al., 2018), of which the mechanism is not clear. It has been shown that implanted extracellular matrix can enhance adipogenesis through local delivery of the pro-angiogenic growth factors VEGF-A, PDGF-BB, and FGF-2 (Ting et al., 2014). PDGF and VEGF in the samples showed significantly higher levels in the DAM group than in the ADM group at different time points, which also indicated that DAM had a better ability to promote angiogenesis *in vivo*. Both groups had similar trends of the growth factors over time, with a gradual increase in PDGF and FGF-2 levels and a gradual decrease in VEGF levels. Some researchers believe DAM stores many growth factors, including VEGF, FGF-2, and PDGF, promoting neovascularization *in vivo* (He et al., 2019; Mohiuddin et al., 2019). It was also attempted to detect the concentrations of growth factors in the decellularized matrices before implantation compared to after implantation, and the proteins in DAM and ADM were extracted using the same method. The detected protein concentrations were too low for the subsequent factor assay, and protein electrophoresis showed almost no significant protein bands (Supplementary Figure S6). This result indicates that after the decellularization process, the decellularized matrix comprises insoluble proteins with collagen as the main component. In contrast, soluble proteins, including growth factors, are present in low amounts, so we believe the growth factors secreted by the cells in the organism play the main role in the tissue remodeling process.

In addition to angiogenesis, inflammation plays an essential role in regulating the process of tissue remodeling (Tilg and Moschen, 2006). When the decellularized matrix was implanted in the body, it first caused the aggregation of inflammatory cells and the release of inflammatory factors. High levels of MCP-1 and MIP-1α were detected in samples in the first week in both groups, compared to weeks 3 and 5, indicating recruitment for macrophages and precursor cells early in the implantation period. Then, the levels of the associated cytokines, such as IL-6, IFN-γ, and TNF-α, increased subsequently. The levels of TNF-α and IFN-γ in the DAM group were higher than that in the ADM group, which may be related to the faster degradation rate of DAM. The degradation of the decellularized matrix induces an inflammatory response, and moderate inflammatory stimulation facilitates cell recruitment and tissue regeneration. As a traditional pro-inflammatory factor, TNF-α protein levels significantly increase in adipose tissue of obese animals and humans (Xu et al., 2003; Lumeng et al., 2007), yet many studies have shown that TNF-α plays a negative role in regulating adipogenesis (Tang et al., 2006; Cawthorn et al., 2007). In addition to immune cells, adipocytes can also secrete TNF-α, so the levels of TNF-α in the implant continued to increase at week 5 in the DAM group.

Transcriptomic profiles of implants revealed divergent transcriptomic patterns between DAM and ADM. In the DAM group, 16 adipogenesis-related genes showed high expression, including *adipoq*, which was consistent with the level of protein ACRP30 in the samples. KEGG gene enrichment analysis showed that highly expressed genes in the DAM group were enriched in several signaling pathways. Among them, the Hippo signaling pathway was one of the significant pathways in the enrichment analysis. Previous studies suggested that it had a bidirectional role in regulating adipose cell proliferation, differentiation, and adipogenesis (Hong et al., 2005; An et al., 2013; Yu et al., 2013b; Huang et al., 2013). *MST*, *LATS*, *YAP*, and *TAZ* are essential regulators of adipocyte proliferation and differentiation in the Hippo signaling pathway. The DAM implant activated *LATS1/2*, which induced *TAZ* phosphorylation and its subsequent inhibition, leading to cytoplasmic retention and the inability to activate the transcriptional activity of *TEAD* in the nucleus. Without *TAZ* in the nucleus, the transcriptional activity of *PPARγ* was increased to promote the expressions of pro-adipogenesis genes (such as *perilipin*, *adipoq*, and *MMP-1*). The expression of MST-related protein *SAV1* was increased in the DAM group, and it was found that *MST1/2* activity promoted direct binding of *SAV1* to *PPARγ*, leading to *PPARγ* protein stabilization and enhanced its lipogenic transcriptional activity (Park and Lee, 2011). In addition, phosphorylated TAZ inhibits the Wnt signaling pathway and exerts lipogenic effects by inhibiting proliferation and promoting differentiation. Both groups showed different degrees of enrichment of the Wnt signaling pathway, induced by the high expression of different Wnt proteins (Wnt5a, Wnt9a, Wnt16), respectively. Wnt signaling is often associated with the control and maintenance of stem cells and can influence the expressions of key transcription factors in adipocyte differentiation. (Han et al., 2019) The primary role of the Wnt/β-catenin signaling pathway is to inhibit mesenchymal stem cell differentiation and increase preadipocytes’ population. (Cawthorn et al., 2012; Chen et al., 2018; de Winter and Nusse, 2021) Concurrent enrichment of both pro-adipogenic and anti-adipogenic signaling pathways in the early stages of DAM implantation suggests that adipose tissue regeneration results from sophisticated regulation of multiple factors in the body. Preadipocyte proliferation and adipocyte differentiation are indispensable in remodeling the decellularized matrix to adipose tissue.

The interaction between the extracellular matrix and the cell is reciprocal and is called “dynamic reciprocity”- the extracellular matrix secreted by the cell can activate cell surface receptors and affect cell growth and differentiation (Schultz et al., 2011). The decellularized matrices from tissues in different health states can build up different microenvironments and continuously send signals to regulate cellular behavior (Hussey et al., 2017; Naranjo et al., 2020), so can decellularized matrices derived from different tissues. However, the generation and transmission mechanisms of such regulatory signals are still unknown. Notably, the decellularized matrix is a unique structure composed of multiple structural proteins, basement membrane proteins, etc., and its biological impact cannot be attributed to any single component or combination of components (Hussey et al., 2017). Therefore, the role played by the spatial structure and composition of DAM in tissue remodeling needs to be further explored. Also, many studies have placed DAM in action in different application scenarios (Mohiuddin et al., 2019), and the influence of the local environment on DAM-induced tissue remodeling should be investigated. Researches on the role of decellularized matrix in promoting tissue remodeling are more helpful in revealing the mechanisms of tissue regeneration and facilitating clinical translational applications.

Finally, the present study used a subcutaneous implantation model to verify the difference in response of the two decellularized matrices. Tissue origin can influence the biological response of decellularized matrix, which may also be a key reason for DAM’s ability to induce adipogenesis. Therefore, the tissue specificity of DAM can be fully utilized for soft tissue repair and regeneration.

## Conclusion

As decellularized matrices of different tissue origin-DAM and ADM, DAM retains the specific properties from adipose tissue after decellularization and exhibits different cellular and tissue responses from ADM in the experiment. *In vitro*, DAM induced adipogenic differentiation of ADSCs, and *in vivo*, DAM implantation led to the release of multiple growth factors and inflammatory cytokines to induce adipose tissue remodeling, fully demonstrating tissue specificity’s effect on the regenerative capacity of the decellularized matrix. In the early post-implantation period, *Lats1/2* regulated adipocyte proliferation and differentiation in implants via the Hippo Signaling Pathway, in conjunction with the Wnt signaling pathway and PPARγ signaling pathway, which co-regulate the adipose tissue remodeling process. Tissue origin plays a vital role in the biological response of decellularized matrix. Further studies are needed to explore the critical factors of DAM-induced tissue remodeling to establish a rational link between its physical properties, molecular composition, and biological functions. Thus, the modulation of the DAM function can be achieved, facilitating the development of tissue engineering and clinical translation.

## Data Availability

The original contributions presented in the study are included in the article/Supplementary Material, further inquiries can be directed to the corresponding authors.

## Abbreviations

ADM, acellular dermal matrix; ADSCs, adipose mesenchymal stem cells; DAM, decellularized adipose-derived matrix; DEGs, Differentially expression genes; DTB, decellularized trabecular bone; ECM, extracellular matrix; eECM, esophageal ECM; GAG, glycosaminoglycans; KEGG; Kyoto Encyclopedia of Genes and Genomes; qRT-PCR, Quantitative real-time polymerase chain reaction.
