# Supplementary material for: Investigating the Adipogenic Effects of Different Tissue-Derived Decellularized Matrices
Source: Front Bioeng Biotechnol. 2022 Apr 14;10:872897. doi: 10.3389/fbioe.2022.872897 (PMC9046558; doi:10.3389/fbioe.2022.872897)
Supplement: Supplementary file 1 [file DataSheet2.docx]

***Supplementary Material***


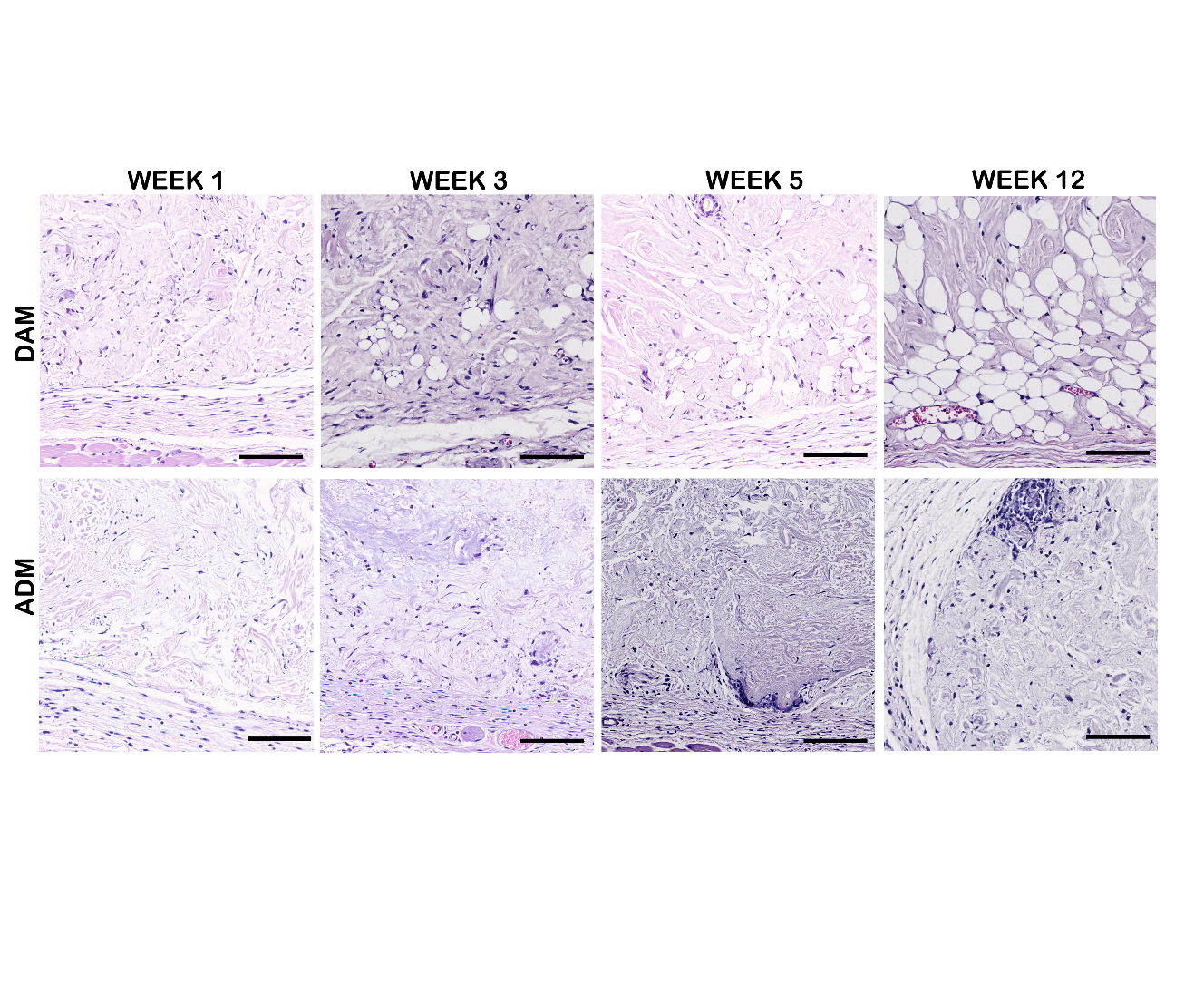
**Supplementary Figure S1.** H&E staining of DAM and ADM implants at weeks 1, 3, 5, and 12. Scale bars =100 μm.


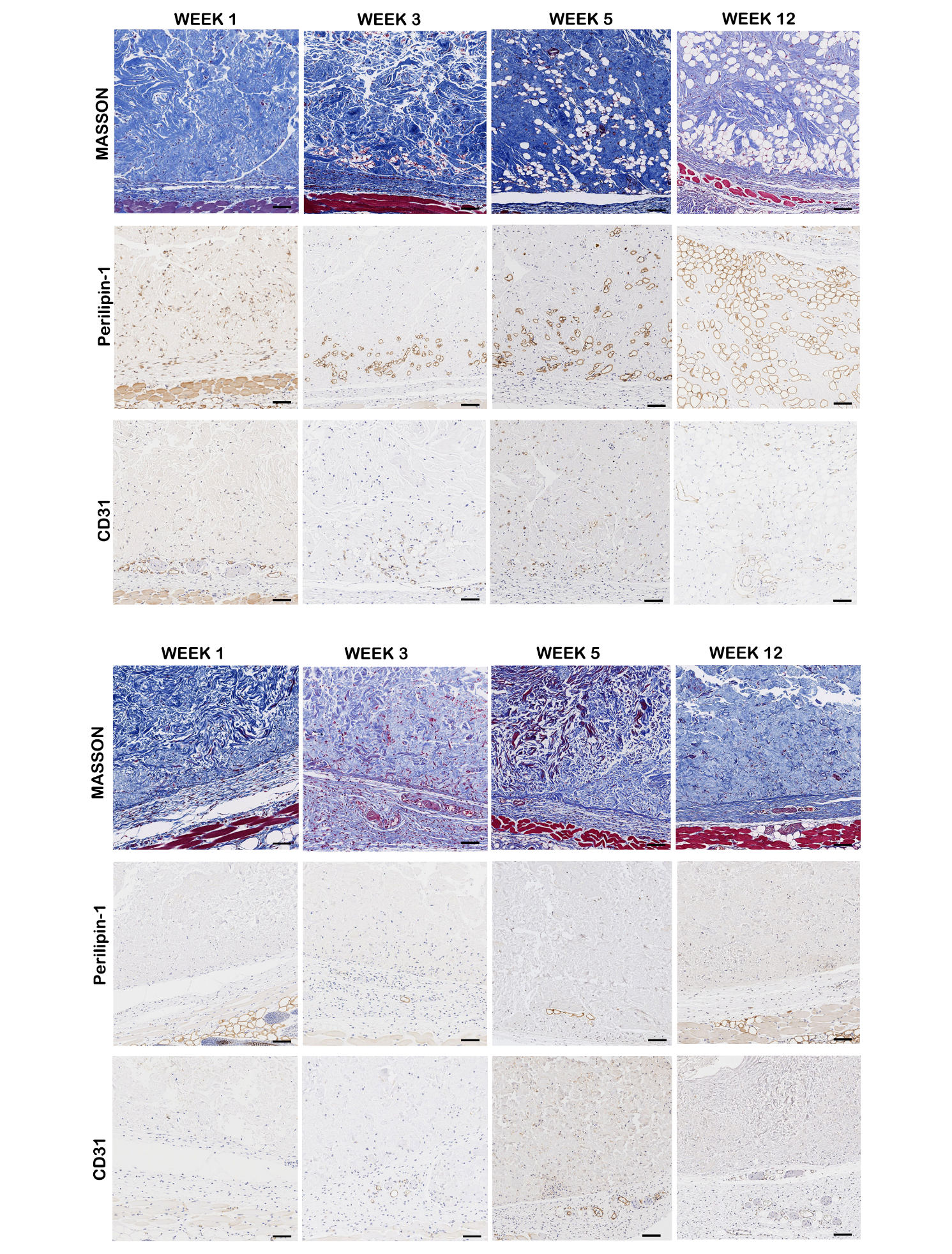


**Supplementary Figure S2.** Masson trichrome staining and Immunohistochemistry of DAM implants at weeks 1, 3, 5 and 12. Scale bars =100 μm.


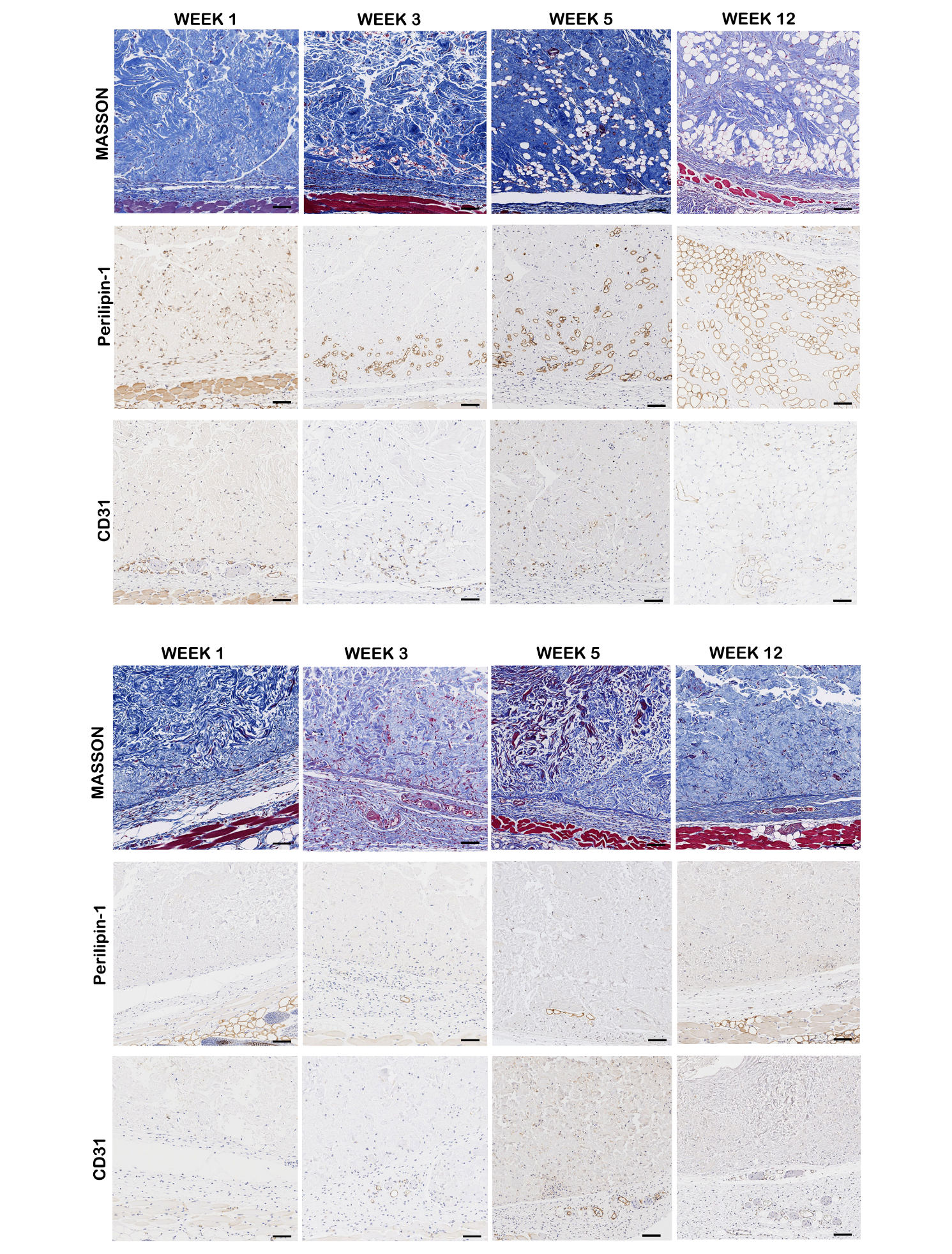


**Supplementary Figure S3.** Masson trichrome staining and Immunohistochemistry of ADM implants at weeks 1, 3, 5 and 12. Scale bars =100 μm.


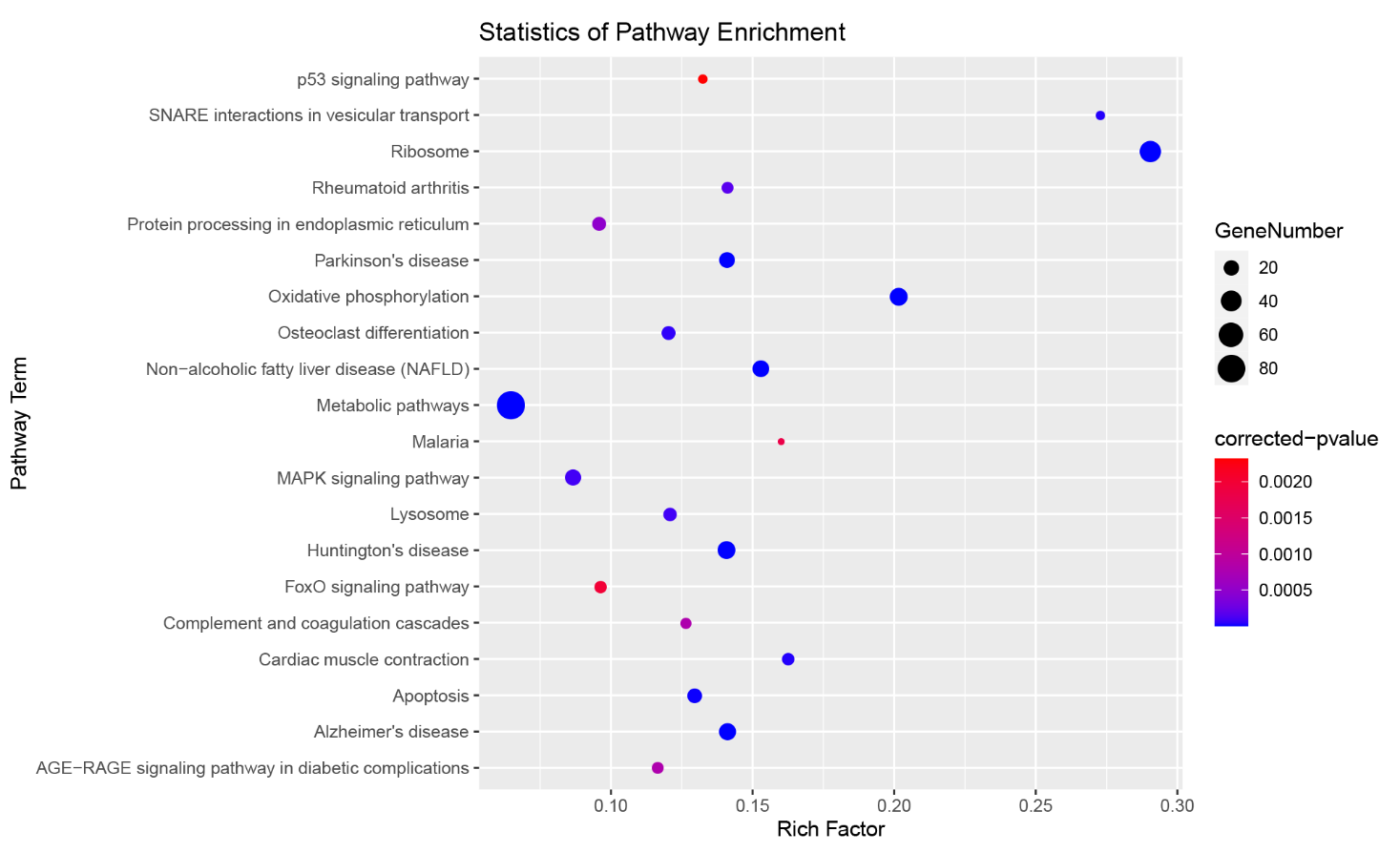


**Supplementary Figure S4.** The top-20 up-regulated KEGG pathways in the ADM group.


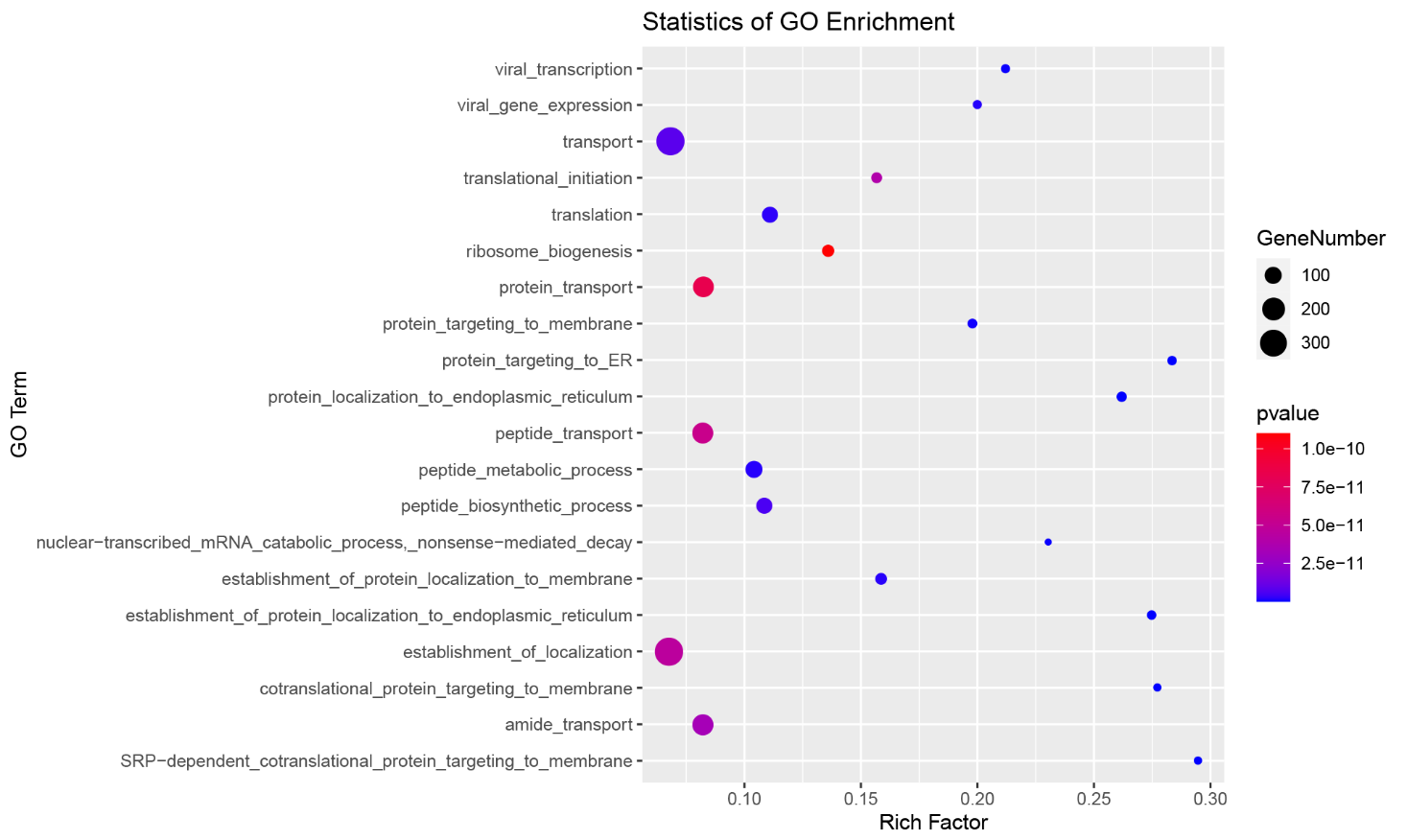


**Supplementary Figure S5.** The top-20 up-regulated GO terms (Biological Process) in the ADM group.


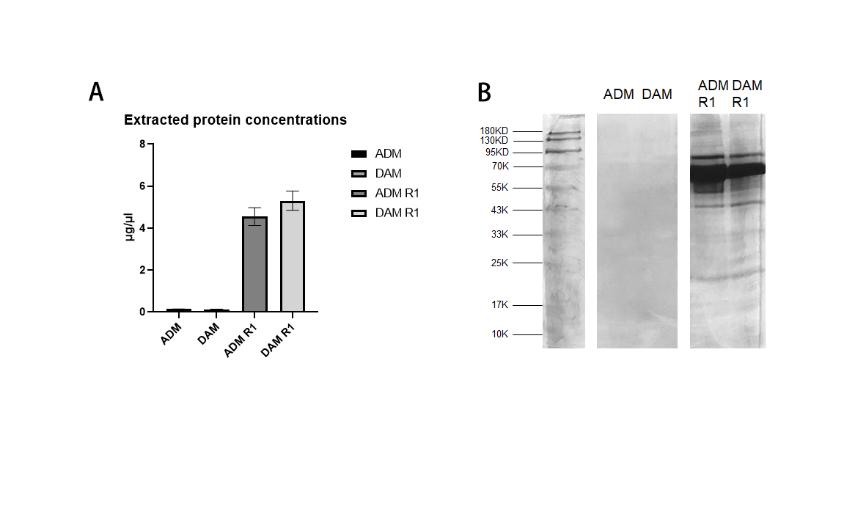


**Supplementary Figure S6.** A. Extracted protein concentrations of ADM, DAM and implants at week 1. B. Protein electrophoresis of ADM, DAM and implants at week 1.

**Supplementary Table S1.** The qRT-PCR primers.

| Primer | Sequence(5’-3’) |
| --- | --- |
| Plin1-F | GCATCGAGAAGGTGGTAGAGTT |
| Plin1-R | TGCTGACCCTCCTCACAAG |
| Adipoq-F | GTGAGACAGGAGATGTTGGAATGAC |
| Adipoq-R | AGTCCCGGAATGTTGCAGTAGAA |
| Wnt5a-F | CAACTGGCAGGACTTTCTCAA |
| Wnt5a-R | CATCTCCGATGCCGGAACT |
| Lats1-F | AATGAAATGATGCGGGTTGGA |
| Lats1-R | CAAACGCTCCTATTCCTAATGTCTT |
| Lats2-F | ATGGTAAAAGGAAACTGGACTAACA |
| Lats2-R | AAACTTCGGGGTGGCTCTCAT |
| Smad3-F | AAAGAACACCGATTCCACTCAACTA |
| Smad3-R | CACCAGAACAGAAGCCATCACTTG |
| Sav1-F | CCCGAGCCCCTGTGAAATATGAC |
| Sav1-R | TGCTTGCGGTTTTCCAACTCAGTG |
| Cebpb-F | CCGGATCAAACGTGGCTG |
| Cebpb-R | CCCGCAGGAACATCTTTAAGTG |
| Wwtr1-F | AGCAACATGGACGAGATGGATAC |
| Wwtr1-R | CAGTAGTGATTACAGCCAGGTTAGA |
| Dlk1-F | GTGCAACCCTGGCTTTCTTC |
| Dlk1-R | TCAGCCTCGCAGAATCCATAC |
| Gapdh-F | AGGTCGGTGTGAACGGATTTG |
| Gapdh-R | TGTAGACCATGTAGTTGAGGTCA |
